# Comparing x-ray phase-contrast imaging using a Talbot array illuminator to propagation-based imaging for non-homogeneous biomedical samples

**DOI:** 10.1038/s41598-023-33788-7

**Published:** 2023-04-28

**Authors:** Mirko Riedel, Kirsten Taphorn, Alex Gustschin, Madleen Busse, Joerg U. Hammel, Julian Moosmann, Felix Beckmann, Florian Fischer, Pierre Thibault, Julia Herzen

**Affiliations:** 1 Chair of Biomedical Physics, Department of Physics, School of Natural Sciences, Technical University of Munich, 85748 Garching, Germany; 2 Munich Institute of Biomedical Engineering, Technical University of Munich, 85748 Garching, Germany; 3 Institute of Materials Physics, Helmholtz-Zentrum Hereon, 21502 Geesthacht, Germany; 4 Institute of Forensic Medicine, Ludwig-Maximilians Universitaet, Munich, Germany; 5 Department of Physics, University of Trieste, Trieste, Italy

**Keywords:** Computational biophysics, Computed tomography, Medical imaging, X-rays, Imaging and sensing, Biomedical engineering, Imaging techniques

## Abstract

Phase-contrast computed tomography can visualize soft tissue samples with high contrast. At coherent sources, propagation-based imaging (PBI) techniques are among the most common, as they are easy to implement and produce high-resolution images. Their downside is a low degree of quantitative data due to simplifying assumptions of the sample properties in the reconstruction. These assumptions can be avoided, by using quantitative phase-contrast techniques as an alternative. However, these often compromise spatial resolution and require complicated setups. In order to overcome this limitation, we designed and constructed a new imaging setup using a 2D Talbot array illuminator as a wavefront marker and speckle-based imaging phase-retrieval techniques. We developed a post-processing chain that can compensate for wavefront marker drifts and that improves the overall sensitivity. By comparing two measurements of biomedical samples, we demonstrate that the spatial resolution of our setup is comparable to the one of PBI scans while being able to successfully image a sample that breaks the typical homogeneity assumption used in PBI.

## Introduction

Over the years, various imaging techniques have been developed, enabling synchrotron imaging experiments to visualize the phase-shifting properties of an object on a microscopic level. The advantages of such techniques include a strongly improved soft tissue contrast^1^. By now, implementations of phase-contrast micro computed tomography (CT) include propagation-based imaging (PBI) techniques^2^, grating-based phase-contrast imaging (GBI)^3^, edge illumination^4^, Hartman-Shack masks^5^ and, as a most recent addition, the speckle-based imaging (SBI) techniques^6,7^, among others. While all these methods are based on the phase-shifting properties of the sample, they differ strongly in terms of experimental requirements and implementation, as well as post-processing algorithms.

PBI techniques are comparably simple to be implemented on an existing setup. The phase-shift can be recovered from a single measurement, given that simplifying assumptions about the sample can be applied, as for instance the single-material assumptions or negligible attenuation^8^. However, if these assumptions are not applicable, phase-retrieval becomes more complex. This can affect compatibility with other investigation methods. Conventional histological investigations use staining to identify functional structures. These stains bind only to specific structures instead of just aggregating in the sample. This approach has been translated to X-ray imaging as well, while maintaining compatibility with visual light microscopy^9,10^. As these stains modify the absorbing properties of the sample, they can interfere with common phase-retrieval assumptions of PBI. For a single-distance PBI phase-retrieval the sample is often assumed to be either transparent or made of a single material. When highly absorbing stains are added, these assumptions can no longer be successfully applied. In contrast, beam modulated differential phase contrast methods, like e.g. SBI, can retrieve the phase signal without any assumptions about the sample. Their disadvantages usually lie in setup complexity or their influence on the spatial resolution by the optical elements.

In recent years, novel speckle-tracking algorithms were developed making SBI a general term for phase-contrast methods using a random wavefront modulator. While implicit tracking methods^11,12^ are more related to PBI, explicit methods^13,14^, like the Unified Modulated Pattern Analysis (UMPA) rely on correlation analysis or cost functions to retrieve the imaging signals and are part of the differential phase-contrast methods. By replacing the random speckle pattern with a periodic structure, e.g. a 2D Talbot array illuminator (TAI) phase grating, the visibility of the wavefront marker can be improved, allowing for more efficient scans^15^.

For biomedical imaging, all of the methods mentioned above are in use. The choice of the suitable setup often depends on multiple parameters. Setup-dependent properties, like complexity, the measurement time, and the computational effort for phase retrieval, differ and have to be considered during the design of an experiment. The exact question on a sample can also require or exclude certain methods, as spatial resolution, phase sensitivity and the ability to gather quantitative information about the sample are dependent on the method. While PBI delivers high-resolution scans, its sensitivity is lower, compared to the differential phase-contrast techniques^16,17^. GBI is able to provides quantitative phase information, however, typically leads to a resolution loss due to the optical elements. For SBI the properties depend on the choice of the tracking algorithm. With the latest addition to explicit speckle tracking algorithms, the Unified Modulated Pattern Analysis (UMPA), SBI has shown its application possibilities for phase-contrast CT at a high spatial resolution^18^ while still providing quantitative data.

**Figure 1:**
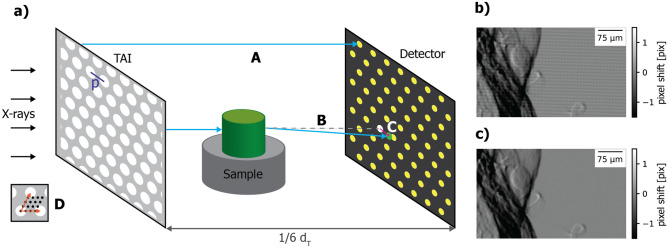
Schematic drawing of the setup in (**a**). The hexagonal TAI with a period $p=10~{\upmu {\hbox {m}}}$, a duty cycle of DC = 1/3, and a phase shift of $\phi =\frac{2\pi }{3}$ is placed with a fractional Talbot distance of 1/6 d$_T$ = 0.54 m (at a beam energy of 20 keV) to the detector. The sample is mounted on the rotation stage in between. A marks a non refracted beamlet. When one beamlet passes the sample as shown in B, it is refracted from its original path (grey dashed line) and the pattern on the detector is shifted as shown by the magenta arrow in C. To record a full scan, multiple rotations are done, stepping the TAI in between rotations along the grating axes in fractions of a grating period, as shown in D. The steps are then used to determine the shift vector for each pixel using UMPA. In (**b**) and (**c**) a comparison of a differential phase image using a mean flat-field image in (**b**) and an optimized flat-field image in (**c**) is shown. The scan used 16 TAI positions and was retrieved using an UMPA window of 3 pix. A total of 15 Eigenflats were used in the optimization. The sensitivity (cf. Eq. 3) of the image in (**b**) is at 500 nrad, in (**c**) at 268 nrad.

In this work, we apply our novel setup (cf. Fig. 1a at the beamline P05 at the storage ring PETRA III, operated by the Deutsches Elektronen-Synchrotron (DESY), using TAIs as wavefront marker in combination with UMPA as phase-retrieval for scanning two biomedical samples. While doing so, we compare the image quality and spatial resolution of the new setup to common propagation-based phase-retrieval, assuming a single material. With scanning a stained soft tissue sample, we show an example of the limitations of the homogeneity assumption of PBI and demonstrate how quantitative phase-contrast methods can have advantages for such cases.

## Results

### Flat-field synthesizing

Compared to previous works, we modified our scan procedure. By switching to continuous rotation scans (fly-scans), the scan-time was decreased. Modifying the flat-field synthesizing process to the usage of eigen flat-field images^19^ improved the sensitivity of individual projections strongly. An example projection is shown in Fig. 1b and c. The scan used 16 wavefront marker positions and an UMPA window of $3\, \times$ 3 pix. In Fig. 1b the differential phase in x-direction is show, when using a mean flat-field image. The background shows residues of the grating structure. In Fig. 1c the same region is shown when 15 principal components were used to synthesize a flat-field image. The background appears smooth. The sensitivity in the projection using the mean flat-field image was at 500 nrad, using the Eigenflat-field images it was at 268 nrad.

### Comparison of TAIs and PBI

The first sample measurement was to compare the resolution of phase-contrast imaging with our TAI-based setup and UMPA phase retrieval to PBI. We scanned a section of human lung tissue from pathology samples of a fatal COVID-19 case. The sample was embedded in paraffin wax and scanned at the imaging beamline P05 operated by the Helmholtz-Zentrum Hereon at PETRA III, DESY. The recorded data were processed for different UMPA window sizes and the resolution at each processing setting was determined. From the same data set a PBI phase-retrieval was computed by averaging over the phase-steps of the TAI scan and reconstructed similarly.

**Figure 2:**
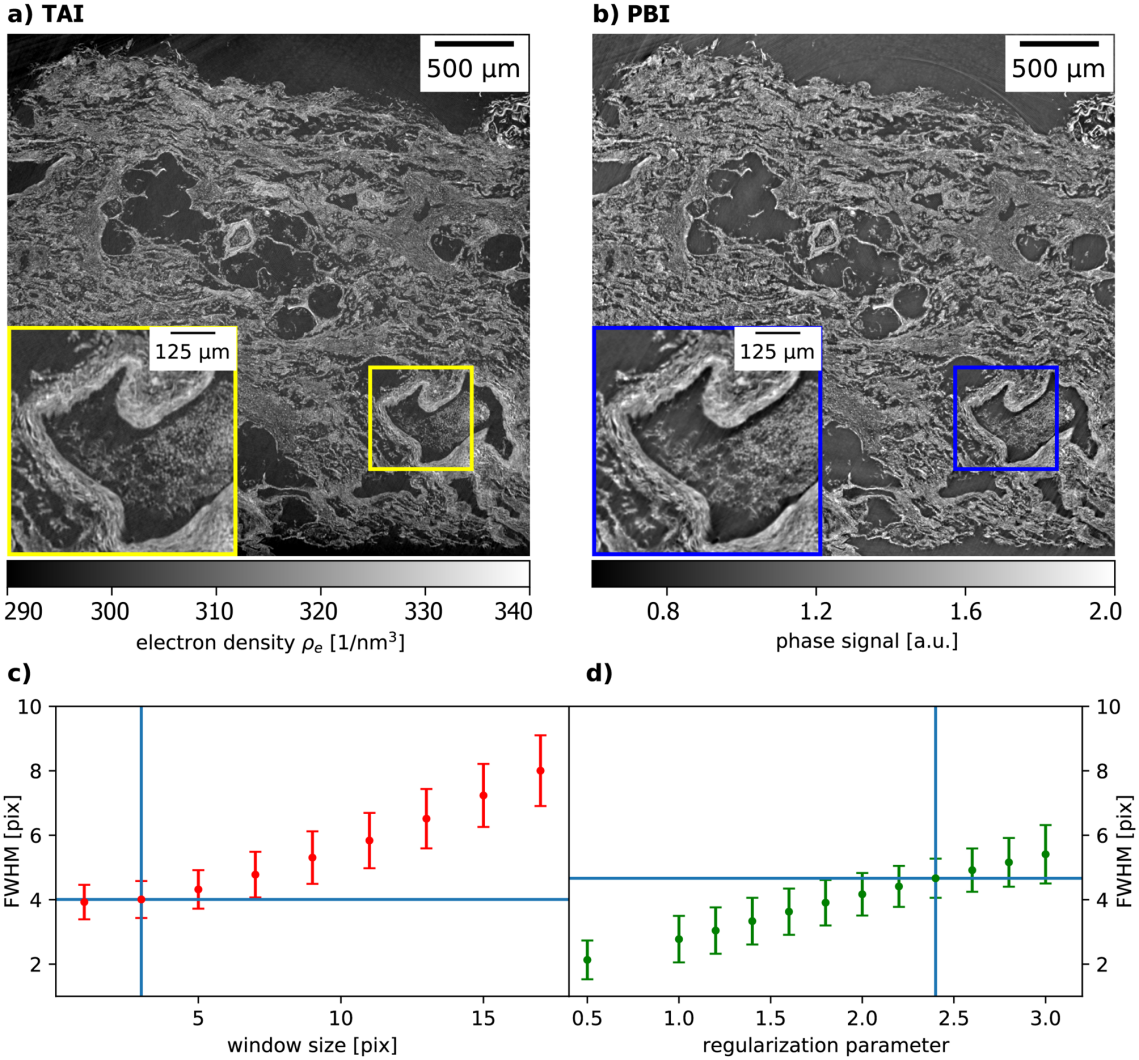
Comparison of a lung tissue sample scan with the TAI and the same data set phase-retrieved using PBI methods. In (**a**) the TAI scan is shown, phase retrieved using UMPA with a window size of $3\,\times$3 pix and converted to units of electron density. In (**b**), the same data set is phase retrieved using a regularized PBI approach with a regularization parameter of 2.4. In plots (**c**) and (**d**), the edge sharpness of both phase-retrieval methods is compared. The edges were fitted with a Gaussian error function fit and the full width at half maximum (FWHM) is being compared. In (**c**) the mean sharpness of several edges are shown for different UMPA window sizes. In (**d**), different PBI regularization parameters are compared. The vertical lines mark the parameters used for the images in (**a**) and (**b**).

The reconstructions with both methods are shown in Fig. 2. In Fig. 2a, an overview of the lung sample is shown, using a $3\, \times$ 3 pix UMPA window. Zoom-ins shows details of blood vessels, with clotted blood in them. The same slice, phase retrieved with a regularized propagation based phase-retrieval is shown in Fig. 2b. A $7\,\times$ 7 pix section of the vessel, was used to determine the Contrast-to-Noise Ratio (CNR) to the embedding material. For the TAI scan this was at CNR$_{TAI}=18.7$, for the PBI scan at CNR$_{PBI}=23.1$.

The slices in Fig. 2 were analyzed for their spatial resolution. Using Fourier ring correlation (FRC)^20,21^, the spatial resolution of the TAI scan was determined to 4.75 ${\upmu {\hbox {m}}}$ at a full-bit resolution criterion and 4.35 ${\upmu {\hbox {m}}}$ half-bit respectively. The resolution of the PBI scan was determined to 4.70 ${\upmu {\hbox {m}}}$ full-bit and 4.15 ${\upmu {\hbox {m}}}$ half-bit resolution. In order to compare the influence of the processing settings, the sharpness of edges in the image were compared for different UMPA window sizes and PBI regularization strengths. This was done by fitting an Gaussian error function (ERF) to the edges. The results are shown in Fig. 2. In Fig. 2c the effect of the UMPA window is analyzed, for window sizes between 1 × 1 pix up to 17 × 17 pix . The slice shown in Fig. 2a used a $3\,\times$ 3 pix window. This resulted in a mean full width at half maximum (FWHM) of the ERF of 4.01 ± 0.69 ${\upmu {\hbox {m}}}$. The regularization strength of the image in Fig. 2b resulted in a mean FWHM of 4.65 ± 0.60 ${\upmu {\hbox {m}}}$. Both phase-retrieval methods show a strong dependence of the edge sharpness on the settings.

### Limitations of PBI

**Figure 3:**
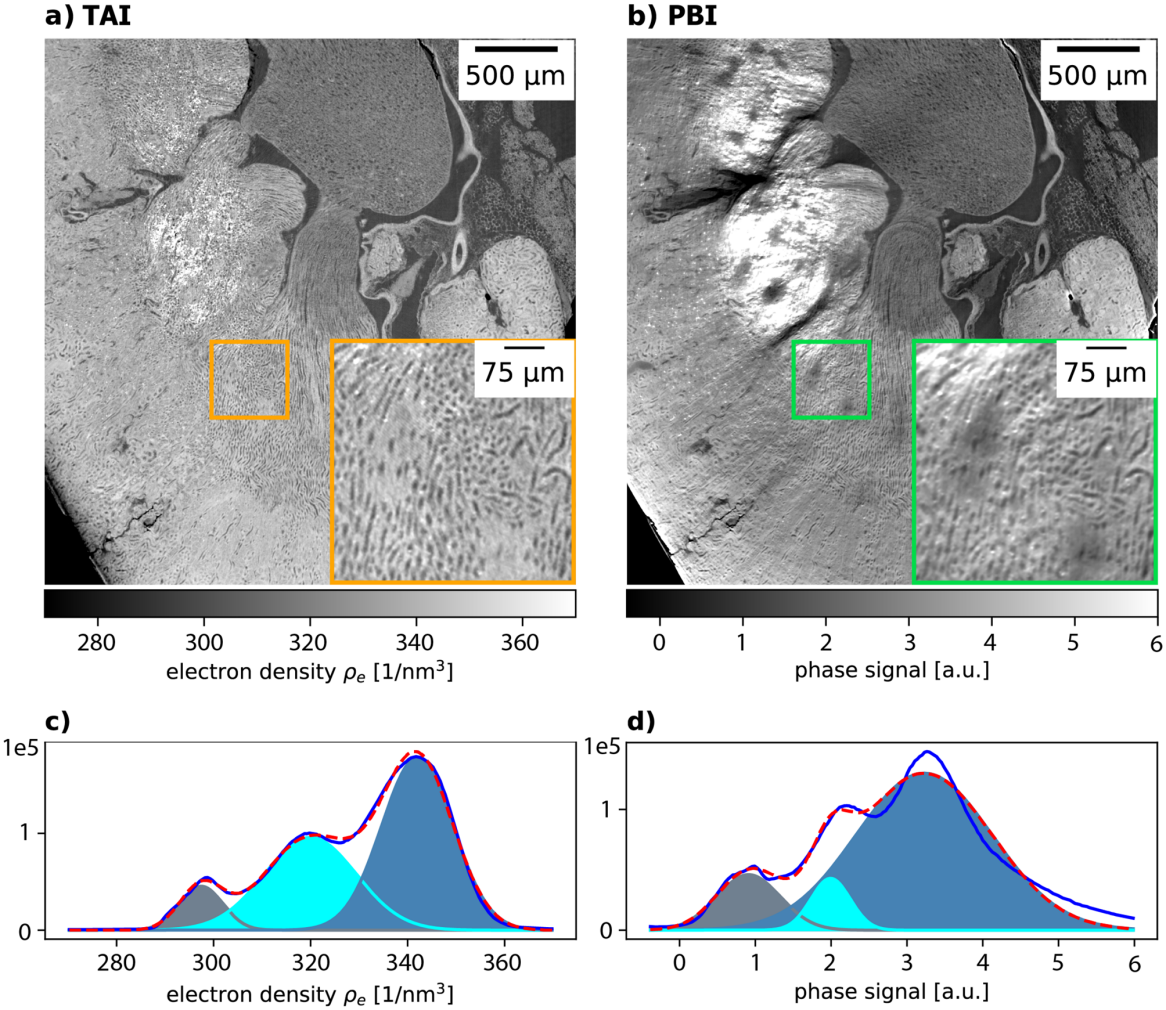
Comparison of quantitative phase-contrast imaging with a TAI in (**a**) with a regularized PBI phase-retrieval in (**b**). The sample was a mouse kidney, which was stained during previous investigation with a Bismuth based stain^22^. Zoom-ins show a part of the outer medulla. The PBI phase-retrieval in plot (**b**) shows strong artifacts where the stain aggregated in the center, as well as towards the edges of the sample. In contrast, the phase-retrieval from the TAI data in (**a**) shows only a slight increase in electron density in the affected areas, but no artifacts. Plots (**c**) and (**d**) compare the histograms of both images. The blue line shows the histogram calculated with 200 bins in the shown window. The red dashed line corresponds to a fit of the histogram using the sum of three Gaussian peaks, which are shown underneath. The peaks correspond to different materials in the scan, from left to right, the gray peak corresponds to the embedding wax, the light blue to the medulla, and the darker blue to the cortex.

In order to exploit the limits of the common PBI assumption of a homogeneous material, we scanned a mouse kidney which was modified with a Bismuth-based stain^22^. The resulting images are shown in Fig. 3a and b. In Fig. 3a the phase-contrast scan using a TAI shows no such artifacts from the stain deposition. The stain aggregation in the center leads to a slight increase in local electron density. In plot Fig. 3b the regularized PBI phase-retrieval is depicted, which shows strong gradients towards the borders of the sample, as well as artifacts in the center.

The histograms of images Fig. 3a and b are shown in Fig. 3c and d. The blue curve shows the histogram, calculated using 200 bins in the shown window. The dashed red line shows a fit of the histogram using the sum of three Gaussian functions, which are depicted individually below. The first peak corresponds to the embedding paraffin, the second peak to the medulla and surrounding tissue, and the third peak to the cortex.

## Discussion

When comparing the phase-retrieval methods for the scan of the lung tissue in Fig. 2, the overall impression is similar for the TAI phase-retrieval and PBI. Some residual edge-enhancement can be found in the PBI images, e.g. inside the vessel walls in Fig. 2b. This indicates a compromise between sufficient regularization strength and not yet dominant blurring. The TAI scan offers a homogeneous image over the whole sample. However, if structural information is desired, both methods offer a similar level of detail. The achieved spatial resolution is for both, the FRC and the edge-sharpness, close for both phase-retrieval methods. PBI offers at the chosen parameter settings a slightly higher CNR than the TAI retrieval.

When PBI and its assumptions about the sample are pushed to their limits, artifacts can be seen in the images. This was the case, when we scanned a stained mouse kidney. The resulting images are compared in Fig. 3. The PBI phase-retrieval in Fig. 3b shows strong gradients towards the edges, as well as artifacts where the stain aggregated in the center. Due to the staining, the absorption behavior is locally varying and the assumption of a single material is no longer applicable. The TAI phase-retrieval (Fig. 3a) in contrast, needs no prior assumptions and can deal with the variations much better. The zoom-ins reveal blurring and inhomogeneities in the PBI scan. The difference is also visible when comparing the histograms of both images. For the TAI scan in Fig. 3c three peaks are visible, which can be attributed to the embedding wax (grey peak), the medulla and surrounding tissue (light blue peak), and the cortex (dark blue peak). Respective peaks can be also attributed to the PBI phase-retrieval data in Fig. 3d, but the ones corresponding to the medulla and the cortex have a shifted position and stronger overlap. The peak of the cortex is much broader, ranging towards higher values. This corresponds to the areas where the stain aggregated and which are now saturated. Compared to the PBI data, the TAI histogram shows a much clearer trimodal distribution of values representing the three domains of the sample.

In our comparison, we take the average over all phase-steps to calculate the propagation image. This blurs out the grating pattern, but can to some extent still produce ring artifacts in the reconstruction. To fully get rid of this, one has to compare two separate measurements, without a modulator in the beam for the PBI scan. As an effect of the uneven illumination by the wavefront marker, the spatial resolution can be influenced. However, if we consider the measured spatial resolution for a low regularization strength, the measurement approached the system resolution. Therefore, the impact of the TAI is considered to be minor. On the other side, the CNR of the measurements can be influenced, as the ring artifacts affect the background noise. Especially for the PBI scan, the determined values can only be seen as a rough estimate of what is possible, as other factors, like increased photon statistics by averaging sixteen scans, can as well affect the values.

The data shown in this work was not recorded at the most optimized configuration of the beamline in terms of spatial resolution. This requires using thinner scintillators, tuning the microscope and camera, and acquiring more projection angles. The limiting factor for the spatial resolution is the light optical microscope of the camera at around 1 ${\upmu {\hbox {m}}}$. This leaves a potential for further resolution improvements if necessary for visualizing a sample.

Compared to conventional GBI setups using multiple gratings, our setup design is easy to implement and requires only one wavefront marker similar to SBI. The usage of a TAI instead of other wavefront markers provides high visibility on a defined length scale and flux efficiency. Combined with the regular nature of the grating pattern, the phase-stepping can be optimized so that fewer steps are necessary to reach a good sensitivity. Unlike random wavefront markers, the periodic structure of the gratings can cause problems if phase wrapping is occurring and can therefore hinder increasing the sensitivity arbitrarily. To avert phase-retrieval problems at edges, the sample can be measured in an embedding medium or the propagation distance can be decreased. The setup was designed for a flexible energy range between 15 and 75keV, addressing a broad class of sample systems from biomedical to material science samples.

The changes in scan recording and processing, made the scans faster, more stable, and more sensitive. Due to continuous rotation scans, the scan duration was shortened to around 2 hours from the previously reported 5 h^15^. A large impact factor on the setup sensitivity is the position stability of the gratings. Our flat-field image synthesizing approach can compensate for wavefront marker or beam drifts to some extent and thus increases the sensitivity to almost half compared to mean flat-field images. This improves the noise in the reconstruction, reduces ring artifacts by reducing static patterns in the phase-retrieved images, and improves the overall image quality. Additionally, the sensitivity of the setup can be tuned for the requirements of the sample by adapting the setup geometry, the number of wavefront marker positions, or the phase-retrieval settings.

For future work, one can exploit the quantitative data from SBI further. Together with quantitative absorption data, material decomposition is possible and can create new insights into the spatial distribution of materials.

## Methods

### Sample preparation

The samples investigated during this work were a piece of human lung tissue and a mouse kidney. The human lung tissue was obtained as an exhibit of a forensics investigation after a COVID-19 fatality by the institute of forensic medicine of the Ludwig-Maximilians university, Munich, Germany. The Ethics Committee of the Ludwig-Maximilians university, medical faculty, waived the need for ethical approval and the need for informed consent (internal reference number 22-0572 KB). All investigations were in accordance with relevant guidelines and regulations.

For the mouse kidney, the animal housing and organ removal was carried out at the Klinikum rechts der Isar, Technical University of Munich following the European Union guidelines 2010/63 and with approval from an internal animal protection committee of the Center for Preclinical Research of Klinikum rechts der Isar, Munich, Germany (internal reference number 4-005-09). After removal, the sample was fixated in a formaldehyde solution and stained with a bismuth-oxo-cluster^22^. After a dehydration series, the stained sample was embedded in paraffin wax and mounted for scanning. All procedures were in accordance with relevant guidelines and regulations, and in accordance with the ARRIVE guidelines^23^.

### Scan parameters

The setup was implemented at the micro-tomography end-station of the imaging beamline P05 operated by the Helmholtz-Zentrum Hereon at PETRA III, DESY^24–26^. An undulator source, in combination with a double crystal monochromator, was used to provide a high coherent monochromatic beam with a photon energy of 20 keV. As the camera, a Ximea CB500MG with a CMOSIS CMV50000 sensor and 7920x6004 pix was used, with a physical pixel size of 4.6 ${\upmu {\hbox {m}}}$. The field of view with a five-fold magnification objective is limited by the beam height to approx. 3 mm, the maximum width at this magnification is 7.29 mm. The spatial resolution of the detector system was determined during the focusing to approx. 1.9 ${\upmu {\hbox {m}}}$. To reach the microscope design limit of approx. 1 ${\upmu {\hbox {m}}}$, the system would have to be adjusted, which includes the usage of thin scintillator screens and closing camera apertures, resulting in a lower light efficiency. This was not done for the scans in this work. A similar setup is available at the High-energy material science beamline P07 at PETRA III for higher photon energies.

Typically used wavefront markers for SBI are sandpaper or steel wool. In order to avoid absorbing elements, phase-shifting grating structures can be used to imprint a pattern onto the wavefront^27,28^. We adapted our wavefront marker to use a 2D phase-shifting grating, a Talbot Array Illuminator (TAI)^15,29^. The grating had a period of 10 ${\upmu {\hbox {m}}}$, a duty cycle of $DC=1/3$, and a phase shift of $\phi =\frac{2\pi }{3}$, with a hexagonal lattice structure. The gratings were manufactured on 200 ${\upmu {\hbox {m}}}$ thick silicon wafers, using deep reactive ion etching to produce round holes with a suited depth. The duty cycle marks the ratio of hole distance to hole radius. At fractional Talbot distances of $\frac{1}{6}d_T$ these TAIs show a focusing effect with a theoretical compression ratio of 1:3 in each direction, thus a high visibility can be achieved. By using TAIs as wavefront markers, absorptive elements were avoided and an efficient two-dimensional stepping could be applied. The periodic nature of such gratings implies an ideal, regular stepping scheme, using a square number of wavefront marker positions. Stepping was performed using a 2D piezo stepper which was directly connected to the detector granite structure for stability. By exchanging the grating and using different etching depths, the setup was already successfully tested at P05 and P07 in an energy range between 15 and 75 keV.

The samples were mounted on an air-bearing rotation stage in between the wavefront marker and the detector. The gratings were mounted 155 mm in front of the sample, the distance from sample to detector was 180 mm for the lung sample and 160 mm for the kidney sample. The total distance varies from the fractional Talbot distance of 538 mm and represents a trade-off between pattern visibility, setup sensitivity, and scan artifact due to edge effects. As edge-enhancement is not included in the UMPA speckle tracking model, too strong fringes cause artifacts at edges and therefore the distance from the sample to the detector is shortened. The distance from the grating to the sample was maximized within the limitations of the setup. The visibility of the wavefront marker was at approximately 0.5. For the given detector resolution, the distance from the samples to the detector is below the critical distance for PBI^30^.

Compared to previous work^15^, the scans were now recorded using a continuous rotation mode. Multiple wavefront marker steps are realized by recording multiple scans, with changing the wavefront marker position in between the scans. A set of 50–100 flat-field images are taken at the beginning and the end of each scan. This improves the scan time and the setup stability, as fewer motors need to be driven.

The scans were performed at a beam energy of 20 keV using 180 degrees rotation. For the lung tissue 4001 projections were taken with an exposure time of 110 ms each while continuously rotating the sample. A total of 16 wavefront marker positions were scanned. The mouse kidney was measured with 2001 projections at 80 ms per image, also with 16 wavefront marker positions. The detector used a 100 ${\upmu {\hbox {m}}}$ CdWO$_4$ scintillator screen and a five-fold magnification objective, resulting in an effective pixel size of 0.92 ${\upmu {\hbox {m}}}$.

### Data processing

The acquired images were corrected for camera dark-current and intensity fluctuations of the beam. As SBI requires reference images to be taken at precisely the same wavefront marker position as the sample projections, a correction for wavefront marker inaccuracies and drifts was required. For this, a principal component analysis (PCA) was performed on the flat-field images of each grating position $f_j$^19^. This yields several so-called Eigenflat-field images $u_k$ and their corresponding Eigenvalues. By using a scree plot, the most important *M* components, in this case 15, were chosen and the remaining components were discarded.

In order to generate a best-fitting reference image at an unknown position of the wavefront marker, a new reference image $f_n$ can be expressed by the averaged reference image $\bar{f_j}$ a weighted sum over the relevant components:

1 $$\begin{aligned} f_n= \bar{f_j}+\sum _{k=1}^M w_k \cdot u_k. \end{aligned}$$

The optimal weights $w_k$ were calculated, by defining a cost function in a background region of the projection $p_{BG}$. Using a least-squares minimization of the difference between the projection and the flat-field image, the set of weights are determined:

2 $$\begin{aligned} w_k=\textrm{arg}\min _{w_k}\left( p_{BG}-f_{n,BG}\right) ^2. \end{aligned}$$

The resulting weights were used to generate the reference image according to Eq. 1. A comparison of the effect of the Eigenflat correction compared to using mean flat-field images can be seen in in Fig. 1b and c.

The projection images and the calculated reference images were then phase-retrieved by using the Unified Modulated Pattern Analysis (UMPA)^14^. The scan was evaluated for varying UMPA window sizes between $1\times 1$ and $17\times 17$ pix. The angular sensitivity $\sigma _{x/y}$, corresponding to the smallest resolvable refraction angle, can be calculated from the noise in a background region, using the standard deviation (STD), of the refraction angle signal:

3 $$\begin{aligned} \sigma _{x/y}=STD(u_{x/y, BG})\cdot \frac{p_{eff}}{d_{prop}}, \end{aligned}$$

where $u_{x/y, BG}$ denotes the calculated pixel shift signal, $p_{eff}$ the effective pixel size and $d_{prop}$ the propagation distance from the sample to the detector.

The resulting differential phase images were corrected for ramps and outliers, anti-symmetrically mirrored^31^, and integrated, using a Fourier approach^32^. After integration, the phase was filtered for ring artifacts and reconstructed via filtered back-projection and a Ram-Lak filter using the software X-Aid (Mitos GmbH, Garching, Germany).

The images for PBI were calculated by taking the mean over all phase steps. Thus the grating pattern is no longer visible, and a regularized approach, based on the Transport of Intensity Equation^8,33^, was used to retrieve the phase. The regularization strength for the slice shown was chosen from visual impression, where no more edge-enhancement was visible inside the sample and blurring was not yet dominant. The projections were reconstructed with the same settings as for the TAI images.

### Resolution analysis

Determining the spatial resolution of the images is not trivial, as multiple effects are present in the images. The raw data contain edge enhancement, whereas the phase retrieved data are blurred to some extend. Therefore we compared multiple methods to for measuring the spatial resolution.

The first method is measuring the edge sharpness. Multiple edges in the reconstructed slices were chosen and an error function (ERF) was fitted. As a resolution criterion, the FWHM of the associated Gaussian of the ERF can be used, which corresponds to approx. $\textrm{FWHM}\approx 2.35\,\sigma$. This method is able to identify blurring efficiently, however the values might be corrupted if edges are enhanced. At the same time, this method is dependent on the sample itself, as is requires sharp edges to be present in the scan.

As second resolution analysis, a Fourier Ring Correlation (FRC) was calculated^20,21^. For this, the image was subdivided in four sub set, by taking every second pixel horizontally and vertically. The FRC was calculated for each of the two diagonal subsets and averaged. The resolution was determined by identifying the intersection of a filtered FRC with a full-Bit and a half-Bit criterion, equivalent to each pixel containing an information content of 1 bit or 1/2 bit respectively. The advantage of this method lies in its independence of the sample. On the other hand, if correlated noise is introduced, the results are corrupted. As phase-retrieval requires processing the raw detector images, the results of the FRC might only be seen approximate for either small UMPA window sizes or small regularization strength.

Other common methods, as e.g. analyzing the Fourier power spectrum^34^ are not suited for determining the resolution of a phase-retrieved image, as they are insensitive to possible blurring introduced in the processing.

## Data Availability

The datasets generated and/or analyzed during the current study are available from the corresponding author on reasonable request.
